# Nanotechnology for Cancer Therapy Based on Chemotherapy

**DOI:** 10.3390/molecules23040826

**Published:** 2018-04-04

**Authors:** Chen-Yang Zhao, Rui Cheng, Zhe Yang, Zhong-Min Tian

**Affiliations:** The Key Laboratory of Biomedical Information Engineering of Ministry of Education, School of Life Science and Technology, Xi’an Jiaotong University, Xi’an 710049, China; zcy18709237339@163.com (C.-Y.Z.); chengrui311@139.com (R.C.)

**Keywords:** chemotherapy, nanoparticles, combination therapy, theranostic nanoparticles, cancer

## Abstract

Chemotherapy has been widely applied in clinics. However, the therapeutic potential of chemotherapy against cancer is seriously dissatisfactory due to the nonspecific drug distribution, multidrug resistance (MDR) and the heterogeneity of cancer. Therefore, combinational therapy based on chemotherapy mediated by nanotechnology, has been the trend in clinical research at present, which can result in a remarkably increased therapeutic efficiency with few side effects to normal tissues. Moreover, to achieve the accurate pre-diagnosis and real-time monitoring for tumor, the research of nano-theranostics, which integrates diagnosis with treatment process, is a promising field in cancer treatment. In this review, the recent studies on combinational therapy based on chemotherapy will be systematically discussed. Furthermore, as a current trend in cancer treatment, advance in theranostic nanoparticles based on chemotherapy will be exemplified briefly. Finally, the present challenges and improvement tips will be presented in combination therapy and nano-theranostics.

## 1. Introduction

Cancer is one of the primary diseases that threaten human lives. Current mainstay treatment of cancer includes surgery, radiotherapy and chemotherapy, among which chemotherapy has been widely performed in clinic because of its simple and convenient process [1,2]. However, there are still some significant limitations in cancer treatment using chemotherapy only. Firstly, chemotherapeutic drugs are nonspecifically distributed in the body and susceptible to multidrug resistance (MDR) during treatment, ineffectively inhibiting tumor growth, metastasis and recurrence [3,4]. Moreover, cancer, which is comprised of various cancer cell subtypes and variable components, can be induced by a variety of carcinogenesis [5]. To overcome these limitations and achieve better cancer therapeutic efficiency, it is necessary to design drug delivery system (DDS) to combine chemotherapy with other cancer treatments. 

The functionalized nano-carriers, which are multidisciplinary products, including medicine, pharmacy, material science and engineering, have been an optimal choice to realize the combination therapy due to their a number of advantages, such as the passive targeting capacity by the enhanced permeation and retention (EPR), the large surface to volume ratio, the ability to load diverse drugs and the tunable surface for targeting modification [6,7,8,9,10,11]. 

Typical nanocarrier systems include liposomes, polymeric nanoparticles, polymer-drug nano-conjugates, dendrimers and inorganic nanoparticles. Each of them displays various unique characteristics. Liposomes, spherical vesicles composed of lipid bilayer with the ability to diffuse across cell membranes easily to deliver agents to cells, can carry hydrophilic and/or hydrophobic therapeutic and diagnostic agents in the inner aqueous core of the structure and/or in the trans-membrane section [12,13]. Polymeric nanoparticles based on synthetic polymers or natural polymers have good biocompatibility with high loading of therapeutic drugs [14]. The multiple surface functional groups of polymeric nanoparticles can also be further modified with some anti-bodies, peptides and other targeting ligands to achieve the active targeting. In addition, the PEG modified on the surface of the polymeric nanoparticles can protect them from the blood clearance by the mononuclear phagocytic system (MPS) [15]. Besides, polymeric nanoparticles can also possess the intelligent responses, such as temperature response [16], pH response [17], redox response [18], light response [19], enzyme response [20] and so on. Polymer-drug nano-conjugates and dendrimers are all based on the advantages of polymer while they have different unique properties. Polymer-drug nano-conjugates, as the name implies, are conjugation that bond polymer with drugs covalently, which increase blood circulation time by escaping filtration from the kidneys. Of note, the increased blood circulation time allows anticancer conjugates to accumulate at the tumor [21]. Dendrimers are polymeric nanocarriers with star-like or branch-like structure, allowing conjugation of therapeutic and/or diagnostic agents on the surface to maximize the effects of cancer treatments [22]. Inorganic nanoparticles, such as mesoporous silica-based nanoparticles, gold nanoparticles and superparamagnetic iron oxide (SPIO) nanoparticles, can be applied into phototherapy against cancer due to their unique photo properties [23,24,25]. All of the nanomaterials mentioned above are good platforms to combine chemotherapy with other treatments of cancer, thereby further improving the efficiency for cancer treatment. At present, a variety of combination therapies based on nanocarriers have been studied, including chemo-chemotherapy combination therapy, chemo-radiotherapy combination therapy [26], chemo-gene therapy combination therapy [27], chemo-photothermal combination therapy [28,29] and chemo-photodynamic combination therapy [30].

Regard to drug loading, one of the imperative concerns for combination therapies based on chemotherapy, there are two main mechanisms to load drugs in nanoparticles: non-covalent interaction and covalent interaction and molecular interactions [31,32]. Because of unique functional groups of drugs and nanocarriers, a variety of drugs can be conjugated to nanocarriers by diverse chemical conjugations, such as ester bond, hydrazone bond and so on [33,34,35]. More importantly, most of chemical bonds will be cleaved at the tumor environment. For instance, Zhang et al. showed that DOX is conjugated to methoxypoly(ethylene glycol)-b-poly(amidoamine) (MPEG-b-PAMAM) by the acid-labile hydrazone, which is easy to cleave under acid environment of tumor sites [33]. Therefore, rational designs of the chemical conjugations can load sufficient drugs and release the drugs at certain times and sites. In addition, drugs can also be encapsulated into nanoparticles through molecular interactions, especially for the hydrophobic-hydrophobic interaction [36]. The methods for drug loading include sonication, dialysis and solvent evaporation. Comparatively, sonication has higher drug loading than the other two methods [37].

Simultaneously, the accurate pre-diagnosis and real-time monitoring are also necessary for cancer treatment, which can provide the sufficient and potent information to design suitable therapy plan [38,39,40]. However, it is easy to delay the best timing of treatment and make patients suffer more pain in traditional clinical application because the diagnosis and treatment of cancer are two relatively independent processes. In order to implement the treatment more effectively and simplify cancer diagnosis, the advent of theranostic nanoparticles with good diagnostic and therapeutic effects has been a new trend in cancer treatment [41,42,43]. Multifunctional nanomaterials can also integrate the diagnosis and therapy in the one nanoparticle with same structure, making the cancer treatment more effective, simple and feasible.

Therefore, it is essential to combine chemotherapy with other cancer treatments or diagnosis. Indeed, increasing studies in this field have been reported. This review summarizes different types of combination therapies based on chemotherapy. Given the increasing importance of diagnosis and real-time monitoring of tumor during cancer treatment, this review briefly presents recent advance in developing theranostic nanoparticles, and then identifies challenges on combination therapies and theranostic nanoparticles from our perspective. 

## 2. Chemo-Chemotherapy Combination Therapy

At present, chemotherapy is one of the most clinically prevailing therapies due to its convenience and there are several chemotherapeutic drugs approved by FDA which have been applied to cancer patients specifically [44]. Considering that cancers are complicated diseases involving multiple pathways and multidrug resistance (MDR) is induced via various and complex mechanisms, several cancers have been treated by combination chemotherapy involving employment of TCH (Taxol, Carboplatin and Herceptin) for HER2/neu-positive tumor and the combination of Platinol (cisplatin) and Navelbine (vinorelbine) for non-small cell lung cancer treatment [45,46]. However, just administrating two or more drugs is not effective because of different pharmacokinetics and biodistribution of drugs [47]. The advent of nanotechnology makes an opportunity to solve the problem. Chemo-chemotherapy combination therapy, using two or more drugs together according to nanotechnology, can maintain the physicochemical properties of the chemotherapeutic agents, which brings about tumor recession more effectively [48,49]. There have been several dual-drug-loaded nanomedicines under preclinical/clinical investigation or clinical trails over the past few years, such as irinotecan/floxuridine, paclitaxel/tanespimycin and cytarabine/daunorubicin [50,51]. 

### 2.1. Chemo-Chemotherapy Combination Therapy Based on Nano-Carriers

**Figure 1:**
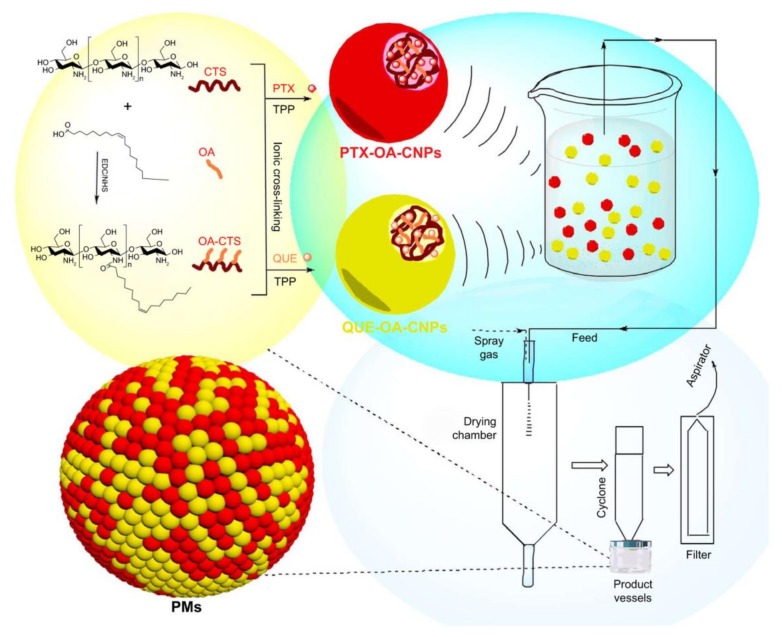
Synthesis and preparation scheme of PMs (PMs, polymeric microspheres; CTS, chitosan; OA, oleic acid; OA-CTS, OA-conjugated CTS; EDC, 1-(3-dimethylaminopropyl)-3-ethylcarbodiimide hydrochloride; NHS, *N*-hydroxysuccinimide; PTX, paclitaxel; TPP, sodium tripolyphosphate; QUE, quercetin; PTX-OA-CNPs, nanoparticles loaded with PTX; QUE-OACNPs, nanoparticles loaded with QUE); reproduced with the permission from [59], Copyright (2017) DOVE Medical Press.

Research reveals that MDR hampers the treatment effects of conventional chemotherapy. MDR is complex due to multiple mechanisms of resistance on a cellular level involving increased activity of efflux pumps, activation of repairing drug-induced DNA damage, break in apoptotic signaling pathways and activation of detoxifying proteins [52]. To reverse MDR, recent studies develop nanoparticles co-delivering chemotherapeutic drugs and MDR inhibitors. For example, P-gp, one of the reasons for MDR, which can limit the anticancer efficacy of drugs due to reduced intracellular drug concentration by pumping out drugs from cancer cells rapidly [53,54,55,56]. Quercetin (QUE), a natural nontoxic flavonoid from numerous vegetables and fruits, has emerged as a P-gp transporter inhibitor [57,58]. Liu et al. designed new NPs in the form of polymeric microspheres (PMs) based on the synthesis of oleic acid-conjugated chitosan (OA-CTS) to co-delivery QUE and conventional chemotherapeutic drug paclitaxel (PTX) (see Figure 1). As QUE inhibits the expression of P-gp transporter and thereby increases the anticancer effects of PTX to the body, this method can prolong PTX retention and release time. In vivo pharmacokinetic and biodistribution studies, PMs exhibited enhanced drugs accumulation and prolonged circulation time in the lung. These results indicated that co-delivery of two chemotherapeutic agents could eliminate cancer cells more effectively by reversing MDR [59]. Dasatinib (DAS), another tyrosine kinase inhibitor targeting various kinases, could reverse the MDR to chemotherapeutic drugs by inhibition of the activation of EPK signaling pathway to downregulate the expression of P-gp [60,61,62,63]. Thus, co-delivery of DAS and doxorubicin (DOX)/PTX based on a simple PEGylated peptidic nanocarrier or simple redoxresponsive micelles increased the intracellular drug concentration by overcoming drug resistance and inhibited tumor growth potently [63,64]. 

**Figure 2:**
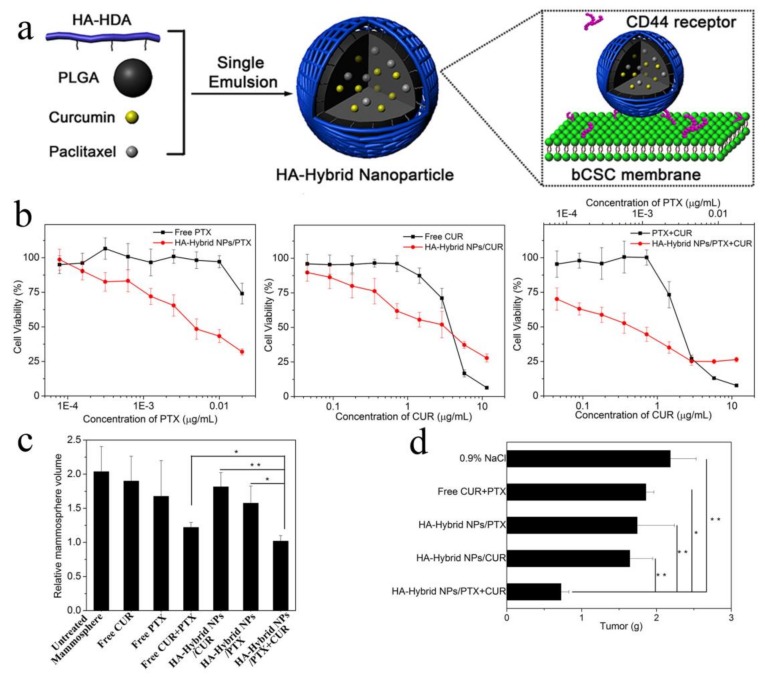
(**a**) Schematic illustration of composition/structure of the HA-Hybrid NPs, and its binding to CD44 on breast cancer stem cells (bCSCs); (**b**) The cell viability of MCF7 mammosphere cells after treated with different drug formulations at varying concentration for 48 h; (**c**) the relative volume of the mammosphere at the end of treatment (volume normalized to that at 0 day); (**d**) the tumor weight at the experimental end point (20th day); reproduced with the permission from [75], Copyright (2017) The Royal Society of Chemistry.

Moreover, recent studies have revealed that tumors comprise a heterogeneous cell population which presents different phenotypic characteristics and proliferative potentials, in which a subset of cells named cancer stem cells (CSCs) attracts more attentions. CSCs can self-renew to form new tumors and consistently proliferate, leading to chemotherapy resistance, relapse, and metastasis [65,66,67]. Therefore, it is vital to simultaneously eliminate cancer cells and cancer stem cells for better cancer therapy, and it is a feasible strategy to use CSCs-targeted chemotherapeutic drugs and conventional cancer cells-targeted chemotherapeutic drugs together. Salinomycin (SAL), a carboxylic acid polyether antibiotic, can eliminate cancer stem cells by activating p38 mitogen-activated protein kinase (p38 MAPK), aggregating reactive oxygen species and inhibiting the Wnt signaling pathway and the activity of P-glycoprotein [68,69,70,71]. Combined use of poly(d,l-lactic-coglycolic acid)-poly(ethylene glycol) nanoparticles of ~140 nm which were separately loaded SAL and chemotherapeutic drug docetaxel (DTX) eradicated both gastric cancer cells and cancer stem cells, suppressing the tumor growth more potently compared with single drug-loaded nanoparticles [72]. Similarly, staurosporine (STS) is a protein kinase inhibitor which eliminates CSCs effectively by inhibiting multiple related signal pathways [73]. The pH-triggered polymeric micellar nanomedicines loading STS and the cytotoxic agent epirubicin (Epi) could simultaneously release both drugs at acidic endosomal pH, overcome the MDR and attain coordinated therapeutic effects to eradicate both CSCs and cancer cells [73,74]. Our group also fabricated a co-delivery system of CSCs inhibitor curcumin (CUR) and PTX to target breast cancer stem cells with attachment of a lipoid (HA-HDA) on the surface of hydrophobic poly(lactic-co-glycolic acid) (PLGA) nanoparticles, inhibiting growth of both breast cancer stem cells and breast cancer cells on MCF7 xenografted tumor model and thereby improving breast cancer therapeutic efficiency (see Figure 2) [75]. Given that fatty acid, nucleic and amino acids are synthesized in cancer cells due to the extensive energy requirement for proliferation, the inhibitors of energy metabolism pathways can be employed to halt the tumor growth, migration and invasion [76]. Phenformin (Phen), as a CSCs inhibitor, interferes with energy metabolism and gemcitabine (Gem), a nucleoside analog and a prodrug, is against most solid tumors [77,78]. Sangeetha et al. combined Phenformin-loaded micelles (Phen M) and gemcitabine-loaded micelles (Gem M), exhibiting higher cytotoxicity compared to Gem M and Phen M alone and significant cell cycle growth arrest in vitro, which obtained better lung cancer therapeutic effects [79,80].

### 2.2. Chemo-Chemotherapy Combination Therapy Based on Carrier-Free Nano-Medicine

Instead of using nanocarriers, a novel carrier-free drug delivery system with high drug loading capacity, no carrier-induced toxicity and simple and green manufacturing process has attracted a lot of attention recently. Carrier-free drug delivery systems are mostly based on amphiphilic drug-drug conjugate to self-assemble into nanoparticles with enhancement of therapeutic efficacy. For example, floxuridine (FUDR) is a hydrophilic therapeutic agent with high antitumor activity against cancer metastases, which can be conjugated with hydrophobic chemotherapeutic agents, such as bendamustine (BdM) and camptothecin (CPT) by ester bond to attain amphiphilic dual-drugs with the ability to self-assemble into stable nanoparticles. In acid environment of tumor cells, the ester bond could be quickly cleaved by hydrolysis to release the drugs, resulting high anticancer efficiency [81,82]. Besides, π-π stacking interactions were applied to assemble DOX and 10-hydroxycamptothecin (HCPT) to form nanostructures, which could also be a good application in carrier-free drug delivery system [83]. 

The molar ration and the physiochemical properties of two therapeutic agents can determine whether the two drugs act synergistically, additively or antagonistically, which can be evaluated by calculating the combination index (CI) [84,85]. CI values lower than 1 indicate synergism, CI values equal to 1 indicate an additive effect, and CI values higher than 1 indicate antagonism. The fact that CI is lower can suggest that the synergism of the two drugs is better, which may reduce the dosage of the drugs and avoid the side effects [75]. More importantly, dual-drug-loaded single nanoparticle may exhibit a better synergistic effect than a simple mixture of two drugs or two individual drug-loaded nanoparticles because that the two drugs in dual-drug-loaded nanoparticles possess similar pharmacokinetics and same delivery targets [86,87,88].

From what mentioned above, one of the key points in chemo-chemotherapy combination therapy is to find the optimized dosage ratio of two drugs, at which the synergistic effect of these drugs can achieve the best results. The optimized molar ratio of cytarabine and daunorubicin is 5:1 in CPX-351, which can achieve maximized therapeutic effects to acute myeloid leukemia [51]. Besides, the change of molar ratio of HCPT to DOX in carrier-free HCPT/DOX nanoparticles results in nanostructures varied from nanorods to nanospheres [83].

## 3. Chemo-Radiotherapy Combination Therapy

Radiotherapy, just as chemotherapy, is one of the most widely used therapies currently, which eliminates cancer cells by radiation-induced DNA damage and cell death [89]. However, the efficiency of radiotherapy is limited by its toxic side effects [90]. Moreover, the simple combination treatment of chemotherapy and radiotherapy also significantly increases the toxicity to patients [91]. To address the limitation, it is a strategy to combine chemotherapy with local radiotherapy synergistically based on biocompatible and biodegradable nano-platforms [90,92]. Thanks to the unique properties of nanoparticles, the chemo-radiotherapy combination therapy can achieve low dose of radiation and low toxicity [90,92].

There are two main categories in chemo-radiotherapy. One is the combination of radiotherapy with chemotherapeutic drugs-loaded nanoparticles. Many researchers have found that liposomes, polymeric nanoparticles and polymer-drug nano-conjugates carrying chemotherapeutic drugs, such as DOX, PTX, DTX and cisplatin could be applicated with radiotherapy to improve therapeutic efficiency. In this method, the nanoparticles usually work as radiosensitizers to sensitize cancer cells to radiotherapy and then the enhanced radiotherapy effects can increase the intracellular concentration of drugs in tumor through EPR [93,94,95,96,97,98].

**Figure 3:**
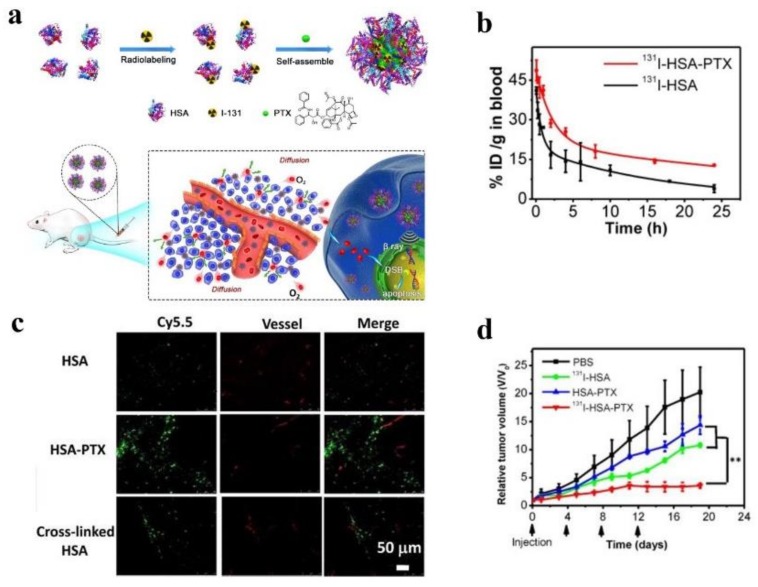
(**a**) A schematic illustration to show the preparation of ^131^I-HSA-PTX nanoparticles for in vivo combined chemo-RIT; (**b**) The blood circulation profiles of ^131^I-HSA and ^131^I-HSA-PT; (**c**) Confocal fluorescence micrographs of tumor slices collected from mice injected with Cy5.5-labeled HSA, HSA-PTX or cross-linked HSA. The red signals were from the fluorescence of anti-CD31-stained blood vessels; (**d**) Tumor growth curves of mice with different treatments given at day 0, 4, 8 and 12; reproduced with the permission from [106], Copyright (2017) Ivyspring International Publisher.

The other one is to develop chemo-radiotherapy nanomedicine which can be co-loaded drugs and therapeutic radioisotopes. The evidence that nanocarriers can be tagged with radionuclides including ^186^Re, ^188^Re, ^64^Cu, ^131^I and ^177^Lu make it possible to co-deliver drugs and radionuclides [99,100,101,102]. It was reported that NPs, composed of a lipid monolayer shell and a polymeric core, could carry DTX and chelate therapeutic isotope ^90^Y to treat ovarian cancer more effectively [97]. Moreover, since human serum albumin (HSA) does not just load hydrophobic diagnostic and therapeutic agents but also be modified and labeled by radionuclides because of the functional groups of HAS, such as carboxyl, amino, phenolic hydroxyl, and thiol groups [103,104,105], one of the recent studies mixed PTX with ^131^I-labeled HAS to develop a nanomedicine, which could prolong blood circulation time, increase the drug concentration in tumor cells and penetrate tumors more effectively compared with any therapy alone, inhibiting the tumor growth significantly (see Figure 3) [106]. In spite of the potent effects of chemo-radiotherapy, the potential toxicity on the normal organs need further preclinical research.

## 4. Chemo-Gene Combination Therapy

Since multiple genetic mutations contribute to tumorigenesis and genetic heterogeneity leads to MDR, therapeutic genes, besides chemotherapeutic agents, have emerged in cancer treatment by precise regulating the specific gene expression, such as MicroRNAs (miRNAs), small interfering RNAs (siRNAs), short hairpin RNAs (shRNAs) and so on [14,107,108]. Among them, miRNAs are small noncoding RNAs, which play an effect on mRNA translation and various biological processes because a miRNA targets multiple mRNAs [109]. Thus, miRNA technique can improve outcomes of cancer treatment by coordinating complicated cancer related signaling pathways and proteins. Apart from miRNAs, siRNA is another potential and potent technique in cancer treatment because siRNAs can selectively silence relevant tumorigenic gene expression through regulation of mRNA translation [110]. However, it is challenging because of nucleases degradation and short circulation time in the blood, poor cell internalization and membrane permeability induced by dense negative charges of nucleic acids and renal clearance [108,111]. Fortunately, combinatorial delivery of genes and chemotherapeutic drugs based on nanotechnology can overcome these obstacles and maximize therapeutic efficiency at the same time [108]. 

Overcoming MDR and regulating cell apoptosis are two main strategies in co-delivery of related therapeutic genes and chemotherapeutic agents. It has been demonstrated that downregulation of P-gp and the major vault protein (MVP) could improve the cancer cells sensitivity to common chemotherapeutic agents and thereby reverse the drug resistance in some cancer cells [112,113]. For example, based on the polymer of *N*-succinyl chitosan-poly-l-lysine-palmitic acid (NSC-PLL-PA), Zhang et al. prepared a triblock copolymer micelle with synergistic effects which could co-deliver antiP-gp siRNA and DOX, significantly increasing the antitumor efficacy in HepG2/ADM cells by downregulating P-gp [114]. Dendrimers co-loaded with MVP siRNA and DOX also showed more cytotoxicity in MCF-7/ADR cells because of the sensitivity of cancer cells to DOX later [115]. In addition, a diversity of cancers including breast cancers, hematologic malignancies and hepatocellular carcinoma, overexpress miRNA21, inducing MDR through repression of the targets of chemotherapeutic drugs, such as PTEN, tropomyosin 1, and PDCD4 [116]. Rui et al. constructed the DOX prodrug (NLS-DOX), condensing anti-miRNA21 electrostatically to form cationic complexes, which were shielded with anionic lipids and Apo AI proteins. This new kind of high-density lipoprotein-mimicking nanoparticles (HMNs) could co-deliver anti-miRNA21 and DOX simultaneously, enhancing the accumulation of DOX in resistant MCF-7/ADR cells and treatment efficiency due to reversal of MDR induced by suppression of miRNA21 expression [109]. Moreover, reports have showed that because of high expression of ABCG2, CSCs exhibited cancer drug resistance by pumping out the internalized drugs [117]. Thus, Chen et al. delivered shABCG2 and DOX synergistically based on a functionalized mesoporous silica nanoparticle (MSN) delivery system, which internalized shRNA efficiently and thereby enriched the intracellular DOX, eliminating cancer cells and CSCs potently [118]. Apart from reversal of MDR, regulation of cell apoptosis-related proteins by relevant genes is also a promising strategy to resensitive the resistant of cancer cells to drugs. Examples that combination of p53 DNA and DOX/PTX or co-delivery of Bcl-2 gene-targeted siRNA and DOX all exhibited increased therapeutic effects because regulation of related gene could enhance drug-induced apoptosis [119,120,121]. Furthermore, the therapeutic effects can be improved by conjugation of targeting ligands against receptors overexpressed in cancer cells. Cyclodextrin-modified dendritic polyamines (DexAMs) were prepared as delivery vehicle to carry anticancer drugs (suberoylanilide hydroxamic acid (SAHA) /erlotinib) and siRNA, which were further modified with targeting ligands. In this way, the toxic side effects on normal cells could be minimized and the proliferation of glioblastoma cells was significantly eliminated due to the synergistic therapeutic effects of chemotherapy and gene therapy [122].

Additionally, antiangiogenesis therapy is another way to treat cancer involving NSCLC. Tumor angiogenesis is driven by vascular endothelial growth factor (VEGF), which is overexpressed in cancer. Blocking the VEGF signaling pathway can suppress the growth of tumor through reduce abnormal angiogenesis in tumor [123]. Thus, siRNA specific to VEGF is a good choice to reduce cancer cell proliferation by decreasing the overexpressed VEGF. For instance, Zhang et al. showed that targeted lipid-calcium-phosphate nanoparticles loaded with gemcitabine monophosphate (GMP) and VEGF siRNA improved therapeutic response supported by eightfold reduction of cancer cell proliferated, significant inhibition of tumor growth and little toxicity in vivo, compared with sole chemotherapy or siRNA therapy in human non-small–cell lung cancer (NSCLC) [124]. 

As mentioned above, it is a promising strategy to eradicate cancer cells and CSCs together. According to recent studies, there are some CSCs-targeted genes that can participate in cancer treatment, such as miRNA-200c [125], miRNA145 [126], miRNA-34a [127] and so on. Here comes an example. Compared with non-CSCs, miRNA-200c is remarkably downregulated in BCSC. Restoration of miRMA-200c can sensitize chemotherapeutic drugs by downregulating classIII β-tubulin [128]. Liu et al. demonstrated that the cytotoxicity of PTX was increased dramatically after miRNA-200c-loaded cationic solid lipid nanoparticles transfected the BCSCs, revealing that chemo-gene combination therapy was a novel strategy in CSCs treatment [125]. 

**Figure 4:**
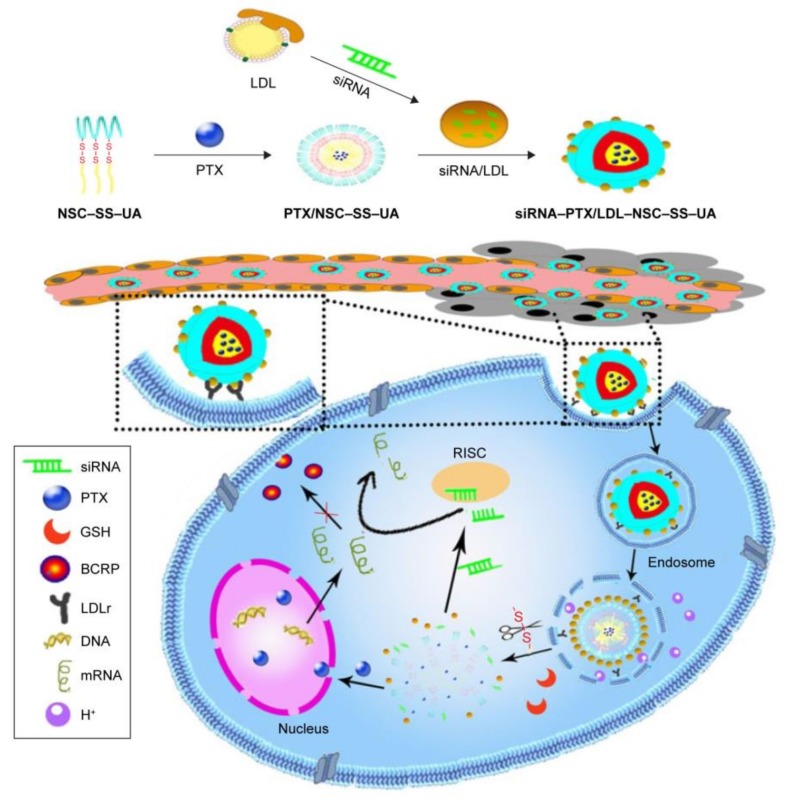
LDL–NSC–SS–UA micelles co-delivering BCRP siRNA and PTX for reversing MDR (BCRP: breast cancer resistance protein; GSH: glutathione; LDL: low-density lipoprotein; LDLr: LDLreceptor; mRNA: messenger RNA; NSC–SS–UA: *N*-succinyl chitosan–cystamine–urocanic acid; PTX: paclitaxel; RISC: RNA-induced silencing complex; siRNA: small interfering RNA); reproduced with the permission from [133], Copyright (2017) DOVE Medical Press.

Furthermore, Tumors are abnormal tissues in an acid environment (tumor tissue: pH 6.5; lysosomal compartments: pH 5.3) with higher concentration of glutathione (GSH) and overabundant enzymes [129]. In order to achieve controlled and predictable therapeutic agents release and to mitigate the side effects to normal tissues, many kinds of stimulus have emerged, such as temperature [130], redox potential [131], pH [132] and so on. Nanoparticles with these stimuli can avoid burst release of encapsulated agents and deliver the therapeutic agents to tumor specifically. Figure 4 exemplified a novel pH/redox sensitive nanoparticles system, which contained nanoparticles (LDL) encapsulating siRNA and polymer *N*-succinyl chitosan–cystamine–urocanic acid (NSC–SS–UA) micelles loaded with PTX. The innovative nanoparticles could overcome MDR and showed higher cellular uptake, better ability to target tumors and improved antitumor capacity [133]. 

More interesting is that there is a possibility that nucleic acids can also be performed as promising nano-carriers thanks to their unique properties. To be specific, DNA possesses the natural ability to self-assemble and conjugate a wide range of molecules, stable structure and programmability and RNA have the properties including thermodynamical stability, biocompatibility and multiple structural flexibility [134,135,136,137]. Moreover, with the ability to integrate a diversity of therapeutic agents, imaging groups and targeting ligands, DNA/RNA nano-carriers have great potential to be applicated in clinic cancer combination therapy.

For a thorough review of chemo-gene combination therapy, we conclude that it is necessary to control the release time of the loaded drug and siRNA. As mentioned above, siRNA is often used to silence the drug resistant phenotype. Therefore, anti-cancer drugs should be released after the drug resistance phenotype is silenced by siRNA to obtain the best synergistic effects. Future studies can explore the strategy to control the release timings of the compounds to maximum the therapeutic efficiency [138]. 

## 5. Chemo-Photodynamic Combination Therapy

Photodynamic therapy (PDT) is a non-invasive cancer therapy with three key players including light source, photosensitizer (PS) and oxygen [139,140]. The common PS for PDT of cancer are chlorins, porphyrins and phthalocyanines, some of which are synthesized to enhance the PDT activity [141,142,143,144,145]. The PS is delivered to tumor sites and is excited after irradiated by light source at a specific wavelength, generating ROS (1O_2_, H_2_O_2_, O_2_*, HO*) and in turn resulting in programmed cell death and repression of tumor growth [146]. Generally, the way of cell death, either apoptotic or necrotic, is determined by the type and concentration of PS, the localization of PS in cells, the type of cell and the light dose [146,147,148]. Moreover, during and after PDT, cancer cells can also be eliminated by inhibiting angiogenesis because that destruction of blood vessels and leakage of fluid and macromolecular in vessels can cause hypoxia locally [149,150,151,152]. 

To enhance the solubility of hydrophobic PSs, reduce side effects to normal tissues and control the release of PSs to maintain the maximized therapeutic outcomes, the application of nanocarriers to immobilize or encapsulate PSs is appealing in the PDT therapy [146,153,154]. Like chemotherapeutic agents, PSs can also be loaded in organic (e.g., liposomes, polymeric nanoparticles, hydrogel) and inorganic nanocarriers (e.g., quantum dots, ceramic-based nanoparticles, carbon materials, metallic nanoparticles) [155,156,157,158,159,160,161,162].

Currently, there have been more interests in the field of chemo-photodynamic combination therapy because the significantly enhanced therapeutic outcomes. The reasons why the combination therapy is better than alone chemotherapy are as follow: (1) the combination of photodynamic therapy and chemotherapy results in synergistic effects due to multiple mechanisms to eliminate cancer cells; (2) PDT can overcome the MDR by decreasing drug efflux via reduction of the P-gp level and then enhance the efficiency of drugs; (3) the generated ROS via PDT can oxidize some nanocarriers, such as phospholipid or cleave thioketal linker to release drugs and enhance the therapeutic effect of nanocarriers-mediated chemotherapy; (4) near-infrared (NIR) can penetrate deeply into tissues of human, leading to eradication of deep cancer cells and local therapy [163,164,165].

The most common method to combine the two therapies is to load chemotherapeutic agents and PSs into one nanocarriers. For example, Habiba et al. designed nanoconjugates (Ag-GQDs/DOX) by conjugating DOX with graphene quantum dots (GQDs)-decorated Ag-NPs against DU 145 and HeLa cancer cells in vitro. The Ag-GQDs/DOX showed high anticancer efficiency by apoptosis in cancer cells without obvious side effects to normal tissues and exhibited ROS-induced cytotoxicity on irradiation at 425 nm. Thus, the Ag-GQDs/DOX with 425 nm irradiation eliminated the cancer cells significantly compared with sole chemotherapy or PDT [163]. Mesoporous silica nanoparticles (MSNs) have large surface area, controllable and tunable pore sizes, so they are one of the potential nanocarriers in cancer treatment. In order to improve the blood circulation time and avoid recognition by the reticule endothelial system (RES), one group developed innovative MSNs-supported red-blood-cell (RBC)-mimetic nanocarriers containing chlorin e6 (Ce6) and DOX. In this system, the RBC membrane stabilized MSNs and improved circulation time in blood by preventing the loaded drugs from leakage. During an external laser source stimulation, Ce6 generated ROS and ruined the RBC membrane and the tumor cell membrane, resulting in a tenfold DOX release and increased DOX accumulation in tumor sites [166]. It is no doubt that MDR is a main obstacle for nanocarriers in cancer treatment, which can also be overcome by chemo-photodynamic therapy. For instance, Shi et al. introduced new photo-activated nanoliposomes (PNLs) consisting of a photosensitizer hematoporphyrin monomethyl ether (HMME) loaded in the phospholipid of nanoliposomes and an antitumor drug DOX encapsulated in the inner chamber of nanoliposomes, which combined chemotherapy and PDT in synchrony. When PNLs were stimulated by a 532 nm laser, a large amount of ROS were generated, causing cell damage, reversal of MDR and oxidation of phospholipid layer which led to rapid release of DOX, further enhancing the antitumor efficiency [164].

**Figure 5:**
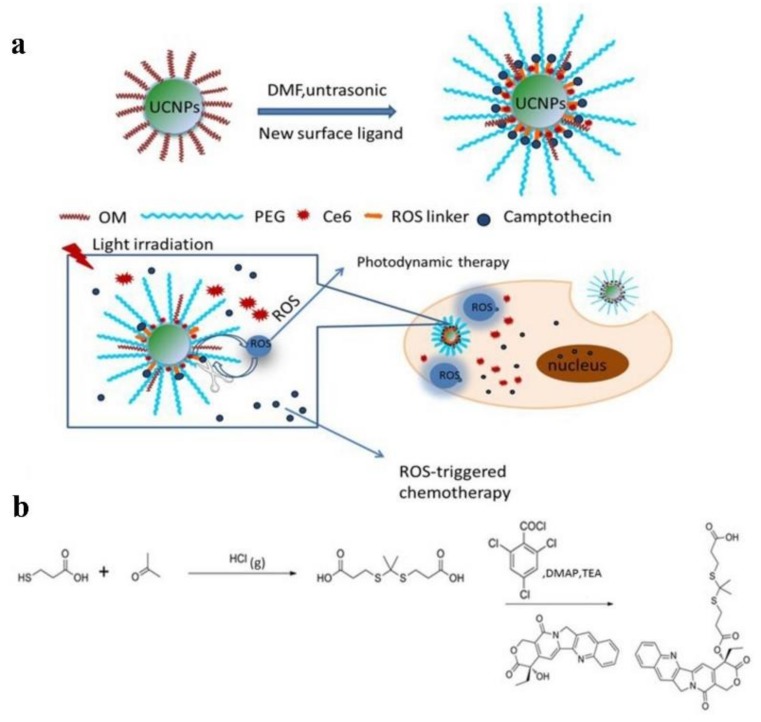
(**a**) Schematic illustration of the preparation of Ce6-CPT-UCNPs and concept of the light-regulated ROS-activated Ce6-CPT-UCNPs, OM: oleylamine; (**b**) Synthesis of ROS-responsive camptothecin, the product was abbreviated as TL-CPT; reproduced with the permission from [165], Copyright (2017) Ivyspring International Publisher.

Besides the synergistic effects in chemo-photodynamic therapy, the drug release can be controlled by taking advantage of the generated ROS in PDT, which means the chemotherapeutic outcomes can be improved via PDT. Thioketal linkage which can be cleaved by ROS, has been widely applied in the ROS-responsive drug delivery systems in chemo-photodynamic combination therapy [167]. A new multifunctional upconversion nanoparticles (UCNPs) system loaded with Ce6, thioketal linker-modified camptothecin (TL-CPT) and mPEG-COOH was shown in Figure 5. UCNPs converted 980 nm light to 645 nm–675 nm light during 980 nm laser irradiation, activing Ce6 and then generating ROS which damaged lung cancer cells and cleaved the thioketal linker in TL-CPT to release CPT for chemotherapy [165]. Similarly, a new mitochondria-targeted drug delivery system, TL-CPT-PEG_1K_-TPP, was synthesized based on polyethylene glycol, TL-CPT and triphenylphosphonium. This system could carry the photosensitizer Zinc phthalocyanine (ZnPc) to form mitochondria-targeted ZnPc/CPT-TPPNPs. Stimulated by 633 nm laser, ZnPc loaded in ZnPc/CPT-TPPNPs produced ROS that was utilized both in PDT and cleavage of the thioketal linker, which caused CPT release for chemotherapy [168]. Other ROS-sensitive linkers, like singlet-oxygen-sensitive labile bis (alkylthio) alkene linker are also utilized in the controlled intracellular drug release and photochemical internalization [169,170]. Interestingly, some types of photosensitizers can also serve as carriers to deliver drugs, which avoid introduction of other organic or inorganic materials. 

## 6. Chemo-Photothermal Combination Therapy

In many combination therapies based on nanotechnology, chemo-photothermal combination therapy is a hot topic recently, mainly due to the fact that photothermal therapy (PTT) is safe and noninvasive. Moreover, upon irradiation of near-infrared laser, PTT does not just convert light energy into thermal energy to increase the temperature of tumor sites to ablate the tumors, but also enhance the chemotherapeutic effects through following aspects: (1) the increased temperature improves the permeability of the cell membrane, accumulating nanoparticles in the cancer cells more effectively; (2) hyperthermia can downregulate the expression of MDR-related genes such as P-gp and MRP, thereby reducing or overcoming MDR in cancer cells; (3) hyperthermia can hinder the repair of DNA damage caused by anticancer drugs in cancer cells, elevating the effects of chemotherapeutic agents [25,53].

Owing to the deeper penetration of NIR, NIR laser is an optimal choice in PTT. NIR photothermal agents include fluorescent dyes (e.g., ICG, IR780), inorganic materials (e.g., carbon nanotubes, graphene oxide and gold materials) and polydopamine (PDA) [171,172,173,174,175]. For example, according to film hydration method, different innovative temperature-sensitive-liposomes were synthesized to realize the chemo-photothermal combination therapy. One was to encapsulate DOX and ICG in the core of liposomes, and the other one was to load DOX in the hydrophilic core and incorporate the liposoluble IR-780 into the lipid bilayer, both of them were proved to be stable in physiological environment and to release more drugs through NIR irradiation at 808 nm, exhibiting laser-controlled drugs release and higher efficiency in tumor inhibition than alone drug-loaded liposomes or fluorescent dyes-loaded liposomes [36,176]. 

In addition, gold nanostructures (e.g., gold nanospheres, nanorods, nanostars and nanocages, nanoshells) have been intensely investigated in chemo-photothermal therapy because of the following reasons. First, under irradiation of appropriate wavelength, gold nanoparticles can convert the absorbed light into local heating, resulting into cancer cells death. Second, it is easy to modify the surface of gold nanomaterials with drugs and other molecules [177,178,179,180,181]. Thus, gold nanoparticles, including gold nanospheres, nanorods, nanostars and nanocages can work as the carriers to deliver chemotherapeutic drugs to tumor sites in chemo-photothermal therapy [24,182,183,184,185]. For example, covalently linked with DOX and cyclic arginine-glycine-aspartic acid (cRGD) to the surface of gold nanostars via GSH, Au-cRGD-DOX was prepared by Chen et al. It was demonstrated that the multifunctional nanoconstruction integrated chemotherapy, photothermal therapy and targeting tumor together and therefore exhibited better treatment effects under NIR irradiation [178]. Besides drugs carriers, gold nanoparticles can also be used as photothermal agents which can be encapsulated into organic nanoparticles like fluorescent dyes [186]. Additionally, it is worth mentioning that a tumor necrosis factor-alpha (TNF) conjugated gold nanoparticle named CYT-6091, which is developed by covalently linking thiol-derivatized poly-ethylene glycol and molecules of TNF on the surface of gold nanoparticle, is in phase II trials in humans [187,188,189]. It was demonstrated that the TNF coated gold nanospheres (Au-TNF) under Laser irradiation showed higher therapy efficacy than Au-TNF alone [190,191]. It opened new avenues for synergistic cancer treatment based on combination therapy.

**Figure 6:**
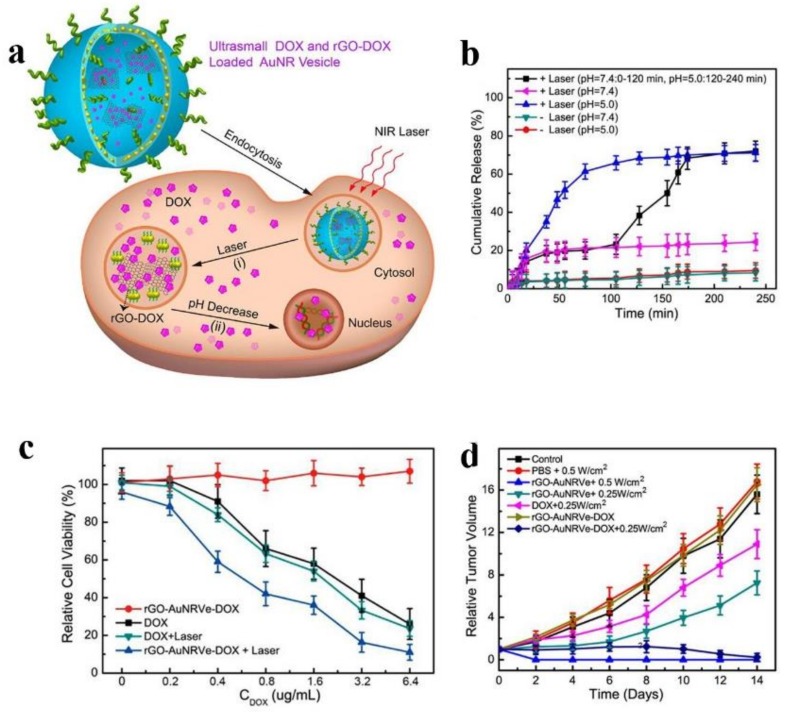
(**a**) Schematic illustration of the sequential drug release in vitro; (**b**) Stimuli-responsive DOX release profiles of different samples irradiated with or without NIR laser irradiation in pH = 5.0 or pH = 7.4 aqueous solution; (**c**) Cell viability of U87MG cancer cells treated with rGO-AuNRVe-DOX and DOX with and without 808 nm laser irradiation at a power density of 0.25 W/cm^2^; (**d**) Relative tumor volume of the tumor-bearing mice after intravenous injection of the samples and exposed to the 808 nm laser at different power densities. Tumor volumes were normalized to their initial sizes; reproduced with the permission from [197], Copyright (2015) American Chemical Society.

Moreover, carbon nanotubes (CNTs) have been considered as a promising photothermal agents and drugs carriers because of their properties including strong NIR photothermal conversion capacity, large surface area and low toxicity [192,193]. It has been reported that various therapeutic agents can be carried by CNTs via π-π adsorption [194,195]. Fu et al. developed a NIR-controlled nanoplatform based on CNTs-oxide nanoparticles, which absorbed DOX and distearoyl-*sn*-glycero-3-phosphoethanolamine-PEG, and then were encapsulated by 1-myristyl alcohol to ensure the stability of the nanoplatform during circulation. When irradiated by 808 nm NIR, the CNTs could absorb the light energy and convert it to heat energy, melting 1-myristyl alcohol and then releasing the loaded drugs quickly. This nanoplatform showed higher antitumor effects both in vivo and in vitro [193]. Similar to CNTs, graphene oxide (rGO) also works as photothermal agent and drug carrier [196]. To increase the photothermal performance of rGO, the plasmonic gold nanorods were modified on the surface to form a new vesicle which could load drugs both in the cavity and on the surface of rGO. After 808 nm laser irradiation, the heat in tumor sites could disrupt the vesicles and then release DOX from the vesicular cavity, and DOX conjugated on the surface of rGO released in intracellular acidic environment. The tumor growth was suppressed effectively in the study due to the enhanced antitumor effects of combination therapy and the sequential and constant release of DOX (see Figure 6) [197].

Under consideration of biocompatibility and biodegradation, a mussel-inspired polymer, polydopamine with strong photothermal conversion ability is appealing in PTT and chemo-photothermal combination therapy. The applications of PDA in chemo-photothermal therapy are classified into three ways: (1) PDA can coat on the surface of drugs-loaded nanoparticles through self-polymerization of dopamine [198]; (2) dopamine nanoparticles act as photothermal agents that are encapsulated in nanocarriers with drugs together [199]; (3) PDA is used as a promising carrier for loading various drugs [200,201]. Attributing to π-conjugated structures and residual phenolic hydroxyl groups of PDA [201,202], it has great potential to utilize PDA in the field of theranostic nanoparticles.

After a thorough review of chemo-photodynamic and chemo-photothermal combination therapies, it is evident that most of the reported nanosystems are complex and they are hard to satisfy the clinical demands due to the various complicated synthesis steps. In addition, non-biodegradable inorganic nanocarriers are widely applied in PDT and PTT. Therefore, it is a tendency to explore nanomaterials with excellent biocompatibility which can be prepared by a simple synthesis. Moreover, light source plays a vital role in PDT and PTT, so it is indispensable to find the ideal wavelength of light [203,204].

Finally, it is worth mentioning that chemotherapy can also be combined with magnetic hyperthermia [205], microwave [206,207], sonodynamic therapy [208,209], immunotherapy [210,211], high intensity focused ultrasound (HIFU) [212] and even be integrated with more therapies, such as chemo-photodynamic-photothermal therapy [213,214,215,216,217,218], chemo-gene-photothermal therapy [219,220], chemo-photodynamic-radiotherapy combination therapy [221], chemo-photodynamic-immunotherapy combination therapy [222] and so on. 

## 7. Theranostic Nanoparticles Based on Combination Therapy and Multimodal Imaging

The applications of imaging techniques in cancer diagnosis and treatment can reflect the states of cancers and the biodistribution of therapeutic agents in real time and in detail. Typical imaging modalities include computed tomography (CT), positron emission computed tomography (PET/CT), single photon emission computed tomography (SPECT), magnetic resonance imaging (MRI), ultrasound imaging (USI), photoacoustic imaging (PA), and fluorescence imaging (FI) [223,224,225]. To boost the imaging contrast, specific contrast agents are applied in different imaging modality. To be specific, contrast agents with high electron density and atomic number, such as AuNPs, barium and iodine compounds are used widely as CT contrast agents [226,227]. Nanoparticles labelled with ^18^F, ^124^I and ^64^Cu or labelled with ^125^I and ^125^Cd can act as PET or SPECT contrast agents to increase the image contrast [223,228,229,230,231]. In addition, for MRI, paramagnetic agents (e.g., Gd^3+^, Mn^2+^, or Fe^3+-^based agents) and SPIONs enhance the contrast of T1 and T2 separately [232,233,234]. Common USI contrast agents are microbubbles which can encapsulate therapeutic agents to realize diagnosis and treatment meanwhile [235]. 

The advent of nanotechnology brings an opportunity to multimodal imaging, which can simultaneously combine two or more imaging modalities to enhance the accuracy of images and exhibit more detailed information of images [212,236,237,238,239,240]. In traditional clinic application, since that the treatment process and the injection of imaging contrast agents are independent, the diagnostic and therapeutic agents cannot be delivered to tumor at the same time, which intensify the pain suffered by cancer patients. To overcome these obstacles, it is a promising clinic application to form theranostic nanoparticles by integrating multiple diagnostic agents with chemo-based combination therapy, which do not just detect the state of diseases, but also deliver therapeutic agents to target sites in control with real-time monitoring of drug pharmacokinetics, distribution and accumulation in tumors, leading to effective tumor inhibition as early as possible. 

There have been a few of studies exploring the application of the multifunctional theranostic nanoparticles. For instance, a vesicle based on rGO named rGO-AuNRVe was mentioned above which was developed by Song et al. The rGO-AuNRVe with ~65 nm could sequentially release loaded drugs via different ways after laser irradiation. Meanwhile, the rGO-AuNRVe with amplified PA performance could be labelled by ^64^Cu, which attributed to the PET application. All in all, rGO-AuNRVe took advantage of synergistic effect of chemotherapy, PDT and PTT and multimodal imaging of PA and PET [197]. In addition, considering the biosafety and biodegradation of organic nanoparticles, an innovative generation of multifunctional poly(vinyl alcohol)(PVA)-porphyrin-based nanoparticles (PPNs) that integrated PET, optical imaging and compound therapy including chemotherapy, PDT and PTT in one formulation could be fabricated through a simple, robust, cost-effective approach. PPNs were proven suitable in vivo NIR fluorescence optical imaging because that they emitted NIR fluorescence with a peak at 680 nm. In addition, ^64^Cu-labeled PPNs were a potential choice to be a positron emission tomography (PET) imaging probe. Moreover, PPNs could prolong the half-life of DOX 53 times in blood circulation and exhibited no obvious cytotoxicity. The properties of PDT and PTT and unique fluorescence self-quenching were showed in the PPNs, which could be a promising method in the field of personalized nanomedicine against cancers [241]. Furthermore, one example of biocompatible nanoplatforms assembling three imaging modalities and multiple therapeutic functions was illustrated by Liu et al. The nanoplatform was synthesized by grafting Fe_3_O_4_ nanoparticles with PEI modified MoS_2_, followed by the load of ICG and platinum (IV) prodrugs on the surface of Fe_3_O_4_ grafted MoS_2_. This theranostic nanoplatform could be loaded with high dose of therapeutic molecules and integrate multimodal bioimaging including MR, PA and infrared thermal with combination therapy involving chemotherapy, PDT and PTT, which was an ideal nanoplatform in future cancer diagnosis and treatment [242]. 

## 8. Conclusions 

The combination therapy, mainly based on chemotherapy and theranostic nanoparticles which integrate multimodal imaging with combination therapy, have been introduced. This field is promising but it still faces some challenges.

First of all, the depth of tumor tissues, with an integral three-dimensional structure, are protected from extracellular matrix and the blood flow is less in the sites, leading to difficult penetration of nanoparticles into the deep of tumor tissues, which restricts the ability of existing theranostic nanoparticles to eradicate cancer cells and CSCs in the deep of tumor tissues. Thus, it is desirable to enhance the penetrating ability of therapeutic nanoparticles via internal and external simulations.

Regard to multifunctional theranostic nanoparticles, since they are loaded and modified with various agents, the complexity of fabrication and possibility of large scale production are barriers which hamper the application in clinic. To solve the problems, tremendous efforts are made to explore robust, simple and cost-effective approach to fabricate multifunctional theranostic nanoparticles that can be utilized in clinic.

Finally, biosafety is the most important issue for patients. However, those organic or inorganic materials introduced in the development of nanoparticles may cause potential long-term toxicity in vivo. In consequence, natural carriers, such as proteins and nuclei acids and no-toxic contrast agents should be introduced to design the nanoparticles. Furthermore, making use of multiple methods to evaluate the toxicity of therapeutic nanoparticles can ensure the biosafety in application.

Considering the fact that the combination therapies can eliminate tumor impressively and increasing researchers focus in this field, we are confident nanodrugs will be increasingly applied in clinic in the coming years.

## Abbreviations and Acronyms

The following abbreviations are used in this manuscript:

MDR	multidrug resistance
DDS	drug delivery system
EPR	enhanced permeation and retention
MPS	mononuclear phagocytic system
SPIO	superparamagnetic iron oxide
QUE	quercetin
PMs	polymeric microspheres
OA-CTS	oleic acid-conjugated chitosan
PTX	paclitaxel
DAS	dasatinib
PEG	polyethylene glycol
CSCs	cancer stem cells
SAL	salinomycin
DTX	docetaxel
STS	staurosporine
Epi	epirubicin
CUR	curcumin
Phen	phenformin
Gem	gemcitabine
Phen M	phenformin-loaded micelles
Gem M	gemcitabine-loaded micelles
FUDR	floxuridine
BdM	bendamustine
CPT	camptothecin
HAS	human serum albumin
miRNAs	MicroRNAs
siRNAs	small interfering RNAs
shRNAs	short hairpin RNAs
MVP	major vault protein
NSC-PLL-PA	*N*-succinyl chitosan-poly-l-lysine-palmitic acid
HMNs	lipoprotein-mimicking nanoparticles
MSN	mesoporous silica nanoparticle
VEGF	vascular endothelial growth factor
GMP	gemcitabine monosposphate
NSCLC	non-small–cell lung cancer
GSH	glutathione
NSC–SS–UA	*N*-succinyl chitosan–cystamine–urocanic acid
PDT	photodynamic therapy
PS	photosensitizer
NIR	near-infrared
GQDs	graphene quantum dots
RES	reticule endothelial system
RBC	red blood cell
Ce6	chlorin e6
UCNPs	upconversion nanoparticles
PTT	photothermal therapy
PDA	polydopamine
GNRs	gold nanorods
CNTs	carbon nanotubes
rGO	graphene oxide
HIFU	high intensity focused ultrasound
CT	computed tomography
PET/CT	positron emission computed tomography
SPECT	single photon emission computed tomography
MRI	magnetic resonance imaging
USI	ultrasound imaging
PA	photoacoustic imaging
FI	fluorescence imaging
DOX	doxorubicin
p38 MAPK	p38 mitogen-activated protein kinase
PLGA	poly(lactic-co-glycolic acid)
HCPT	10-hydroxycamptothecin
HMME	hematoporphyrin monomethyl ether
MPEG-b-PAMAM	methoxypoly(ethylene glycol)-b-poly(amidoamine)
CI	combination index
DexAMs	cyclodextrin-modified dendritic polyamines
SAHA	suberoylanilide hydroxamic acid
cRGD	cyclic arginine-glycine-aspartic acid
TNF	tumor necrosis factor-alpha
Au-TNF	TNF coated gold nanospheres
